# Consistency of Multi-Month Antiretroviral Therapy Dispensing and Association with Viral Load Coverage among Pediatric Clients Living with HIV in Mozambique

**DOI:** 10.3390/tropicalmed9070141

**Published:** 2024-06-26

**Authors:** Ivete Meque, Nicole Herrera, Michelle M. Gill, Rui Guilaze, Amancio Nhangave, Jaciara Mussá, Nilesh Bhatt, Mahoudo Bonou, Lauren Greenberg

**Affiliations:** 1 Elizabeth Glaser Pediatric AIDS Foundation, Maputo, Mozambique; imeque@pedaids.org (I.M.);; 2 Elizabeth Glaser Pediatric AIDS Foundation, Washington, DC 20005, USA; 3 Núcleo de Pesquisa Provincial de Gaza, Provincial Health Directorate, Gaza, Mozambique; 4 Núcleo de Investigação Operacional de Inhambane, Provincial Health Directorate, Inhambane, Mozambique

**Keywords:** HIV, ART, multi-month dispensing, pediatric, Mozambique, viral load coverage

## Abstract

With the increase in uptake of multi-month antiretroviral therapy dispensing (MMD) for children, little is known about consistency of MMD receipt over time and its association with virological outcomes. This analysis aims to assess the uptake of 3-month MMD among children, consistent receipt of MMD after uptake, and clinical outcomes following transition to MMD in 16 health facilities in Gaza and Inhambane Provinces. This is a secondary analysis involving children <15 years living with HIV with clinical visits during the period from September 2019 to August 2020. Of 4383 children, 82% ever received MMD (at least one pickup of a 3-month MMD supply) during the study period but only 40% received it consistently (defined as MMD at every visit during the study period). Consistent MMD was most common among older children and children without indications of clinical instability. Overall viral load (VL) coverage was 40% (733/1851). Consistent MMD was significantly associated with lower odds of having a VL (0.78, 95% CI: 0.64–0.95). In conclusion, while receipt of a multi-month supply was common particularly during the early days of the COVID-19 pandemic, only a minority of children received consistent MMD; however, there is a need to ensure children with fewer visits still receive timely VL monitoring.

## 1. Introduction

In Mozambique, children under the age of 15 years old constitute 6% of all people living with HIV (PLHIV). In 2022, with an estimated 150,000 children living with HIV (CLHIV) in the country, only 73% were receiving antiretroviral therapy (ART) and of those, 71% achieved viral load suppression (VLS) [1]. After the World Health Organization (WHO) endorsed the *Test and Treat* policy in 2015 [2], recommending initiation of ART for all PLHIV regardless of immunologic or clinical status, the global number of PLHIV receiving ART increased exponentially from 17 million people in 2015 [3] to 29.8 million in 2022 [4], a growth of 43% in just seven years. However, this massive increase in the number of PLHIV on ART was not observed among CLHIV aged 0–19 years, who experienced an increase from 43.5% in 2016 to only 57.1% in 2022 [5].

The benefits of the *Test and Treat* policy and early ART initiation in reducing AIDS-related deaths have been widely demonstrated [4]. However, there were concerns that this policy would pose a huge burden to already stressed health systems, particularly in developing countries, in providing quality and efficient health care to large numbers of people on treatment [6,7,8]. As part of innovative and differentiated service delivery, in 2016, the WHO recommended multi-month dispensing models (MMD) aiming to simplify service delivery to PLHIV in the era of *Test and Treat* [2]. These models are designed to be client-centered and to reduce unnecessary burdens on clients and the health system [9]. Extending the interval between ART refills for clinically stable clients (e.g., from monthly to every 3 or 6 months) decongests clinics while reducing the number of clinic visits, time spent at clinics, travel costs, and hours of work and school lost for the client [10,11,12,13]. Adopting such an approach to HIV care ensures that the needs of the diverse groups of PLHIV are tailored to improve access to services and quality of care while effectively allocating the available resources [14]. The clinical outcomes are also positively impacted by MMD. The findings from observational studies and clinical trials also suggest non-inferiority of three- and six-monthly community and facility-based dispensing of ART on retention and VLS compared to standard-of-care monthly facility-based ART delivery [15,16,17,18]. 

However, most studies of MMD models—and most MMD models themselves—have initially focused on adult clients. A number of concerns—difficulties in achieving viral control, the need to adjust regimen and/or dose as a child grows, and a desire to monitor children for other health conditions—have often been barriers to expanding access to nontraditional care models such as MMD.

Mozambique released MMD guidelines in April 2018 allowing for the offer of multi-month dispensation with a three-month supply (3MMD) for ART to clients ≥2 years old, provided they were clinically stable [19]. Clinical stability was defined as having been on ART for at least 6 months (or 12 months for children < 10 years old), having no conditions associated with WHO stage III or IV, and no recent history of adverse medication reactions or poor adherence. The candidates for MMD were also required to have had a VL < 1000 copies/mL within the last year, or, if VL was not available, to have had a CD4+ count ≥ 200 cells/μL (children < 5 years old without a VL were required to have either a CD4+ cell count > 750 or CD4% > 15% to be considered stable) [19]. If children receiving 3MMD experienced viral rebound (>1000 copies/mL), low CD4, conditions associated with WHO stage III or IV, or showed psychosocial problems that impacted adherence, clinicians were advised to replace the 3MMD with monthly ARV dispensing (and enhanced adherence visits, if indicated) until the child was able to return to 3MMD. Children were only eligible for the MMD model if their caregiver was also enrolled in the model and met the criteria for adult MMD participants [19].

In 2020, the SARS-CoV-2 (COVID-19) pandemic provided an opportunity for countries to expedite the implementation of MMD models to protect PLHIV [20], especially for children and others who did not meet strict clinical stability criteria for MMD [21]. Starting in March 2020, many African countries accelerated simplification of MMD eligibility to include children, recommending its rapid implementation to reduce clinic visits and limit PLHIV exposure to SARS-CoV-2, while maintaining a continuous and reliable supply of ART, refocusing facility-based services to care for sicker clients (including those with COVID-19), and relieving pressure on healthcare workers. 

Although data suggest that enrolment into MMD did not compromise clinical outcomes such as VLS among African children and adolescents aged 2–19 years [22,23,24,25], and may have improved retention in care among adolescents and young adults [26], there is evidence indicating that younger children aged 2–4 years were generally less likely to receive MMD [22,27]. Some of the reported barriers for younger children included low stocks of ARV medication due to challenges in the supply chain (particularly for younger children on lopinavir-based regimens) [28], providers fearing to offer MMD to young children due to the need of dosage adjustment as weight increases, and caregiver concerns regarding their children accessing MMD [22]. 

With the increase in uptake of MMD for children, there is a need to better understand the feasibility and outcomes of these models in children and adolescents, particularly as implemented by national HIV care and treatment programs rather than as controlled trials. This study aims to describe the uptake of 3MMD of ART among children under the age of 15 years in Mozambique, the extent to which these children are receiving consistent dispensing of a multi-month supply, and the relationship of multi-month dispensing with selected clinical outcomes in Gaza and Inhambane Provinces.

## 2. Materials and Methods

### 2.1. Study Design, Setting, and Population

The data used for this secondary analysis were collected as part of an observational retrospective cohort study utilizing routinely collected client-level data at 16 health facilities in Gaza and Inhambane provinces in Mozambique. As viral load (VL) outcomes were critical to the study objectives, sites with higher than average pediatric client volume and higher than average pediatric VL coverage (≥60% coverage, compared to average coverage of ~50% in these provinces according to 2019 programmatic data) were purposively selected for inclusion in the study. Ten facilities from seven districts were included from Gaza Province as well as six facilities from five districts in Inhambane Province.

This study abstracted data on all CLHIV aged 0–14 years who had an HIV-related clinical visit to one of these 16 health facilities during the period September 2019–August 2020 (enrollment period). This included children already enrolled in HIV care and treatment services prior to the enrollment period, as well as those newly enrolled in services. There were no exclusion criteria.

### 2.2. Data Collection

Data were abstracted from both electronic and paper-based clinic record sources in three rounds beginning in April 2020 and ending in January 2022, with no direct interaction with clients. Trained research assistants abstracted information on demographics and selected clinical/HIV-related history (date of initiation on ART, regimen at initiation, etc.). Data were also abstracted on each HIV clinical visit during the period from September 2019 to August 2021 (the study period); this included information on ART pickups, VL sample collection dates and results, selected comorbid conditions, death, and other care outcomes. During the process of data abstraction and analysis, research assistants and other members of the study team were able to consult with facility clinicians and other staff to address issues of missing or discrepant data.

### 2.3. Measures and Statistical Analysis

The data on ART pickups were used to create measures related to the uptake and consistency of MMD:

Receipt of MMD: For each ART pickup during the study period (all visits from September 2019 to August 2021), data collectors recorded the amount of ART received as either 30 days (1 month) or 90 days (3 months). Any child with at least one ART pickup of 90 days was classified as having received MMD, and the earliest date on which they received 90 days was considered their date of first receipt of, or transition to, MMD.

Consistent receipt of MMD: Among children with at least 6 months of ART pickup data after first receiving MMD, we categorized children according to their subsequent ART pickups during the following 12 months. If all ART pickups were coded as 3-month receipt, the child received consistent MMD. If not, the child was considered to have received inconsistent MMD (either a combination of 30- and 90-day pickups, or only 30-day pickups at all subsequent visits within that year).

Data from VL monitoring was used to assess VL coverage and suppression as follows:

VL coverage: We defined VL coverage as a record of having any VL result (based on specimen collection date) within the 12 months after first receipt of MMD (not including the date of first MMD), among children with at least 12 months of study follow-up after first receipt of MMD. This is consistent with the national guidance at the time that children should receive VL monitoring 6 months after initiating ART or switching to a new ART regimen but may transition to annual VL monitoring if there was no suspicion of treatment failure [29].

Viral suppression: VL results of <1000 copies/mL (including undetectable) were considered suppressed.

Data analysis was conducted using SAS version 9.4. Statistical comparisons were made using the chi-squared test. Odds ratios and 95% confidence intervals were presented for both unadjusted and adjusted logistic regression models.

### 2.4. Ethical Considerations

This study and the waiver of informed consent were approved by the National Bioethical Committee for Heath of Mozambique and Advarra Institutional Review Board in the United States.

## 3. Results

### 3.1. Baseline Characteristics and Follow Up

Of the 4383 children enrolled in the study, 1183 children (27.0%) were under the age of 5 years old at the time of enrollment into the study, 1699 (38.8%) were aged 5–9 years, and 1501 (34.2%) were aged 10–14 years (Table 1). Male children represented 47.4% of the study population, and more than half of the children had been on ART for over two years at the time of enrollment. Most children (69.7%) had been initiated on a nevirapine (NVP)-based regimen; this was most common among children aged 5 years and older, most of whom had been on ART for more than two years at the time of enrollment. Half of those initiated on NVP had started ART prior to 2016.

Most children enrolled in this study (84.3%) were still active in care at the end of the study period. Slightly more than ten percent of children transferred out to a different facility during the study period, and 3.1% of children were categorized as having defaulted or lost from care. Of the 94 children who died during the study period (2.1% of the total study population), 70 (74.5%) were under the age of 5 years old at the time of enrollment into the study.

### 3.2. Receipt of Multi-Month ART

Over the course of the study period, 82.3% of children enrolled in the study (3609/4383) received at least one ≥90 day supply of ART. This was highly and significantly correlated with age; 86.6% of children aged 5–9 years had received a multi-month supply compared to 62.9% of children aged 0–4 years (OR 3.8, 95% CI: 3.2–4.6), and the proportion was even higher (92.8%) among children aged 10–14 years (OR 7.6, 95% CI 6.1–9.6 compared to children aged 0–4 years). Despite guidelines indicating that children under the age of 2 years old should return monthly to pick up ART, 98 children first received MMD prior to the age of 2 years old. Of these 98, 50 (51.0%) first received MMD during the period March–July 2020 when changes to dispensing related to COVID-19 were first introduced. While there was some variation by site in the proportion of children who ever received MMD, 15 of the 16 sites reported that at least 70% of children had ever received MMD.

**Figure 1:**
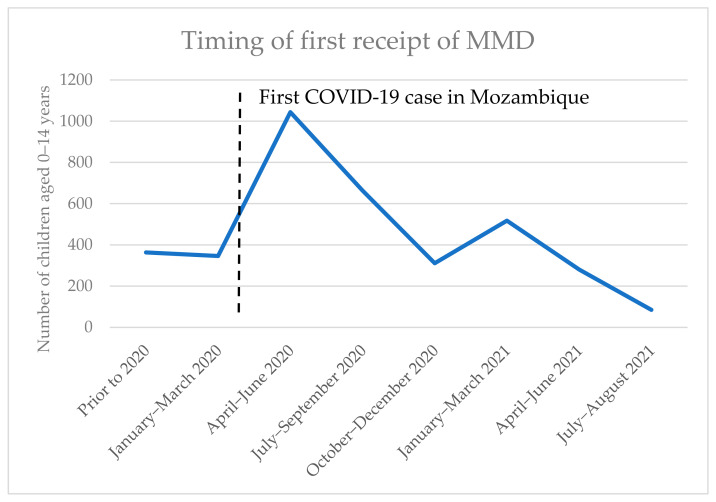
Timing of first receipt of MMD among children 0–14 years.

Among children who ever received MMD during the study period, most (2727/3609, 75.6%) first received MMD before the end of December 2020. Figure 1 charts the number of children transitioned to MMD over time; there is a large increase around the time that COVID-19 mitigation measures were introduced in 2020, and another increase in January–March 2021 when COVID-19 cases in Mozambique rose significantly.

### 3.3. Consistent Receipt of Multi-Month ART

**Figure 2:**
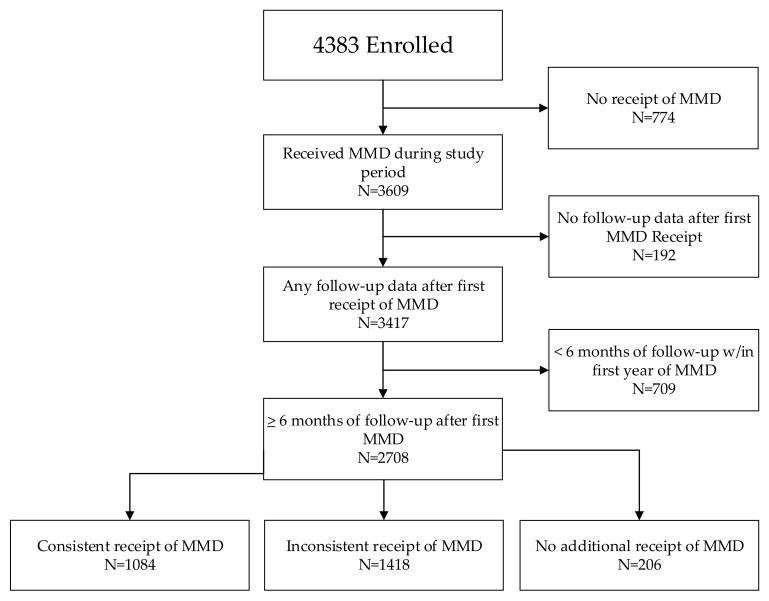
Consistency of MMD receipt after first MMD among children 0–14 years.

Consistent receipt of MMD was analyzed for all children with at least 6 months of follow-up data after MMD transition (N = 2708). To do so, we reviewed ART pickup data for all visits within the first 12 months after first receiving ≥90 days of ART. Overall, 40.0% of children (1084/2708) consistently received a ≥90-day supply of ART at all visits within 12 months of first receiving MMD (Figure 2). Just over half of all children had inconsistent receipt of MMD (at least one pickup each of ≥90 days of ART and of <90 days of ART), and 7.6% of all children (and 17.4% of children under age 5 years) had no record of further receipt of MMD after the first MMD receipt.

We examined a number of factors in relation to the consistency of MMD receipt during the study period, including selected clinical outcomes: VLS, opportunistic infection (OI), and malnutrition (Table 2). These data were only collected for the time period of this study and thus provide a limited representation of the overall client history. However, these measures may have affected clinician decisions about ART dispensing for children. Among clients who received MMD during the study period, 15.3% of children had a documented OI during the study period, and 11.0% had a diagnosis of malnutrition. Nearly all of these occurred after the first receipt of a multi-month supply; fewer than 1% had a record of OI or malnutrition prior to first MMD. While only 70.6% of children transitioned to MMD had a pre-transition VL result available within the study period, 31.8% of these children with pre-transition VL had a history of unsuppressed VL prior to transition.

In adjusted analysis, consistent receipt of a multi-month supply of ART (compared to inconsistent or no further receipt of ART) was significantly associated with older age, receiving care at one of the facilities in Inhambane province, and having no documentation in the patient file or other facility/electronic records of an opportunistic infection or an unsuppressed viral load result during the study period (Table 3).

### 3.4. Viral Load Coverage and Multi-Month ART

We calculated VL coverage as the proportion of children with at least 12 months of follow-up after initiating MMD (N = 1850) who had a VL result within those 12 months (not including the date of first MMD receipt). The overall coverage was 39.6% (733/1850). We further explored the relationship between VL coverage and consistent receipt of MMD through modeling (Table 4). In a model adjusted for province, age group, and sex, consistent MMD receipt was negatively associated with VL coverage.

## 4. Discussion

This study aimed to assess the transition to a 3-month supply of ART among children aged <15 years, consistency in receiving the 3-month supply, and clinical outcomes within the 12-months post-transition. While efforts to broaden access to multi-month ART dispensing models (and other types of differentiated services) pre-date the COVID-19 pandemic, mitigation measures resulted in changes to practice and/or guidelines that dramatically increased the number of children receiving MMD. Mozambique was no exception; the data from our study corroborate other reporting that indicates a significant increase in the number of children who received a 3-month supply of ART starting in 2020. This includes a small but notable number of children who may not have met even the updated criteria for receipt of MMD, such as those under the age of 2 years old, or with a recent history of elevated VL or OI. This study also highlights the gaps experienced by CLHIV on ART in Southern Mozambique in maintaining a consistent 3-month supply at all clinical visits and VL coverage and suppression.

In particular, with the increased focus on transitioning clients (both adults and children) to MMD in countries supported by the Presidents Emergency Plan for AIDS Relief (PEPFAR), the data on the number and proportion of PLHIV transitioned to an MMD model are widely available in many settings. In contrast, little information is available—among adults or children—about ART dispensing patterns following an initial receipt of a multi-month supply. Our study underscores the need for more of this type of monitoring and analysis to fully understand the impact of the MMD model on both client outcomes and healthcare facility capacity.

We found that only 40% of children received a 3-month supply of ART at all subsequent visits during the 12 months after first starting MMD. A number of factors may contribute to inconsistent MMD receipt among children, particularly during this time period of 2020–2021. In addition to issues with ART drug stock shortages (often more challenging for pediatric regimens and formulations, and exacerbated by pandemic-related supply chain disruptions), certain contraindications to MMD, such as elevated VL, are more common among children. The study period also coincided with the rollout of new pediatric drug formulations and treatment guidelines, during which time children who had been receiving a 3-month supply were offered a single month only of the new regimen before returning to the 3-month supply after an acceptability assessment. Other analyses of this cohort found that 88% of children aged 5 years and older had at least one regimen switch during the study period [30]. These switches may have contributed to more frequent clinical monitoring. We also found that children with evidence of an opportunistic infection, a diagnosis of malnutrition, or an elevated VL measure were less likely to receive consistent MMD. These clinical findings may have prompted clinicians to temporarily suspend the 3-month dispensing among children and were in line with guidelines for MMD dispensing, but more information would be needed to fully understand any causal relationships.

Our study also found that older children, particularly those aged 10–14 years, were more likely to receive a 3-month supply than younger children. This is consistent with previous findings that showed that children younger than the age of 9 years old were less likely to be offered MMD than older age groups [22,31]. While MMD is not recommended for children <2 years to facilitate accurate dosing, exclusion of younger children (i.e., ages 2–9 years) from MMD is also a missed opportunity as uptake of MMD allows for more child-centered visit frequency and better alignment with the visit schedules of older family members on ART receiving care through the MMD model. More work is needed to better understand the barriers that preclude uptake of MMD by these children under the age of 10.

A concerning finding from this study is the low VL coverage. VL monitoring is a critical component of HIV care and treatment, particularly for children who are less likely to achieve consistent viral suppression compared to adults in most settings (including in Mozambique). The (negative) association of VL coverage with consistent receipt of MMD merits further investigation and attention to ensure that children transitioned to this model continue to receive necessary services, whether that is VL monitoring or other services not assessed through this evaluation, such as TB screening or psychosocial support. Our study thus highlights the need for continuing efforts for inclusion of younger clinically stable children into differentiated ART models while maintaining continuous VL monitoring in accordance with national guidelines.

This study is subject to limitations, many of which are common to the secondary analyses of routine service delivery (non-research) data. Despite routine data quality assurance activities at health facilities, supplemented by further quality checks by the study team, we encountered a number of cases of missing or discrepant data, which were resolved to the extent possible by the study team. A few factors that may have impacted clinicians’ dispensing decisions were either not captured through the study or not recorded consistently enough in facility records for analysis, such as indications or concerns of sub-optimal adherence, or the need for additional services (such as psycho-social support), that would indicate the need for more frequent return visits to the health facility. We also collected limited data on client clinical and service utilization history prior to the study period, meaning that we may have underestimated the incidence of certain outcomes (such as history of OI or elevated VL) or underestimated the time on MMD for a small number of children who may have first received MMD prior to September 2019. The enrollment of children at different points during the study period, the variation in amount of follow-up time and data available for analysis after first MMD, and the large number of children who transferred out of a study site prior to the end of data collection may have caused us to underestimate the true number of children transitioned to MMD.

## 5. Conclusions

While a significant proportion of children enrolled in HIV care and treatment programs were offered MMD in 2020–2021, only about half of these children continued to benefit from this model—whether due to client/family preference, loss of eligibility for MMD, drug availability/stockouts, or clinician decision. More information is needed to better understand the causal relationships between these factors and how they impact or are responsive to ART dispensing practices. Low VL coverage continues to be a significant challenge that may be exacerbated by the reduction in clinic visits by children on consistent MMD, and should be prioritized for further investigation by researchers and program implementers.

## Figures and Tables

**Table 1:** Demographics and HIV-related history.

	Age at Study Enrollment			
	<5 Years N (col%)	5–9 Years N (col%)	10–14 Years N (col%)	Overall N (col%)
Total N	1183	1699	1501	4383
Sex				
Male	564 (47.7)	837 (49.3)	676 (45.0)	2077 (47.4)
Female	619 (52.3)	862 (50.7)	825 (55.0)	2306 (52.6)
Regimen at ART initiation				
Missing	2 (0.2)	8 (0.5)	6 (0.4)	14 (0.3)
Efavirenz-based	28 (2.4)	100 (5.9)	213 (14.2)	341 (7.8)
Nevirapine-based	556 (47.0)	1352 (79.6)	1147 (76.4)	3055 (69.7)
Lopinavir-based	567 (47.9)	160 (9.4)	42 (2.8)	769 (17.5)
Dolutegravir-based	14 (1.2)	55 (3.2)	74 (4.9)	143 (3.3)
3 NRTIs	15 (1.3)	24 (1.4)	16 (1.1)	55 (1.3)
Other	1 (0.1)	0	3 (0.2)	4 (0.1)
Time on ART at Study Enrollment				
<6 months	464 (39.2)	200 (11.8)	194 (12.9)	858 (19.6)
6–<12 months	125 (10.6)	82 (4.8)	61 (4.1)	268 (6.1)
12 months–<24 months	212 (17.9)	170 (10.0)	217 (14.5)	599 (13.7)
≥24 months	382 (32.3)	1247 (73.4)	1029 (68.6)	2658 (60.6)

**Table 2:** Consistency of MMD receipt within 12 months after transition, among children with at least 6 months of follow-up ART data.

	No Additional MMD N (row %)	Inconsistent MMD N (row %)	Consistent MMD N (row %)	Total
Total N	206 (7.6)	1418 (52.4)	1084 (40.0)	2708
Province				
Gaza	149 (7.4)	1133 (56.6)	721 (36.0)	2003
Inhambane	57 (8.1)	285 (40.4)	363 (51.5)	705
Sex				
Male	106 (8.5)	663 (52.9)	484 (38.6)	1253
Female	100 (6.9)	755 (51.9)	600 (41.2)	1455
Age group				
<5 years	78 (17.4)	241 (53.8)	129 (28.8)	448
5–9 years	85 (8.1)	593 (56.3)	375 (35.6)	1053
10–14 years	43 (3.6)	584 (48.4)	580 (48.1)	1207
Documented opportunistic infection during study period				
Yes	54 (13.0)	225 (54.3)	135 (32.6)	414
No	152 (6.6)	1193 (52.0)	949 (41.4)	2294
Documentation of malnutrition during study period				
Yes	32 (10.7)	166 (55.5)	101 (33.8)	299
No	174 (7.2)	1252 (52.0)	983 (40.8)	2409
Documentation of unsuppressed VL during study period				
Yes	67 (11.1)	349 (57.9)	187 (31.0)	603
No (including those with no VL data)	139 (6.6)	1069 (50.8)	897 (42.6)	2105

**Table 3:** Factors associated with consistent receipt of MMD (compared to inconsistent receipt of MMD or no further receipt of MMD).

	Consistent MMD N (row %)	Inconsistent/ No Further MMD N (row %)	Total	Odds of Consistent Receipt of MMD within 12 Months of First MMD		
				Unadjusted OR (95% CI)	Adjusted OR (95% CI)	Adjusted *p*-Value
Total N	1084	1624	2708			
Province						<0.0001
Gaza	721 (36.0)	1282 (64.0)	2003	REF	REF	
Inhambane	363 (51.5)	342 (48.5)	705	1.89 (1.59–2.25)	1.88 (1.57–2.25)	
Age group						<0.0001
<5 years	129 (28.8)	319 (71.2)	448	0.73 (0.58–0.93)	0.75 (0.58–0.95)	
5–9 years	375 (35.6)	678 (64.4)	1053	REF	REF	
10–14 years	580 (48.1)	627 (51.9)	1207	1.67 (1.41–1.98)	1.58 (1.33–1.88)	
Sex						0.331
Male	484 (38.6)	769 (61.4)	1253	REF	REF	
Female	600 (41.2)	855 (58.8)	1455	1.12 (0.96–1.30)	1.08 (0.92–1.27)	
OI reported						0.005
Never reported	949 (41.4)	1345 (58.6)	2294	1.46 (1.17–1.82)	1.39 (1.10–1.75)	
Ever reported	135 (32.6)	279 (67.4)	414	REF	REF	
Malnutrition reported						0.067
Never reported	983 (40.8)	1426 (59.2)	2409	1.35 (1.05–1.74)	1.28 (0.98–1.65)	
Ever reported	101 (33.8)	198 (66.2)	299	REF	REF	
VL ≥ 1000 copies/mL						<0.0001
Never reported (including children with no VL data)	897 (42.6)	1208 (57.4)	2105	1.65 (1.36–2.00)	1.58 (1.29–1.93)	
Ever reported	187 (31.0)	416 (69.0)	603	REF	REF	

**Table 4:** Model of VL coverage within 12 months after first receipt of MMD.

	Had VL N (row %)	No VL N (row %)	Total	Odds of VLC within 12 Months of First MMD		
				Unadjusted OR (95% CI)	Adjusted OR (95% CI)	Adjusted *p*-Value
Total N	733 (39.6)	1117 (60.4)	1850			
Province						0.758
Gaza	569 (39.9)	857 (60.1)	1426	REF	REF	
Inhambane	164 (38.7)	260 (61.3)	424	0.95 (0.76–1.19)	1.04 (0.83–1.30)	
Age group						0.0002
<5 years	111 (42.4)	151 (57.6)	262	REF	REF	
5–9 years	290 (45.8)	343 (54.2)	633	1.15 (0.86–1.54)	1.17 (0.87–1.56)	
10–14 years	332 (34.7)	623 (65.3)	955	0.73 (0.55–0.96)	0.76 (0.57–1.01)	
Sex						0.552
Male	332 (39.1)	517 (60.9)	849	REF	REF	
Female	401 (40.1)	600 (59.9)	1001	1.04 (0.86–1.26)	1.06 (0.88–1.28)	
Ongoing receipt of MMD						0.013
Consistent MMD	261 (35.3)	478 (64.7)		0.74 (0.61–0.90)	0.78 (0.64–0.95)	
			739			
Inconsistent/No further MMD	472 (42.5)	639 (57.5)	1111	REF	REF	

## Data Availability

The datasets used and/or analyzed during the current study are not publicly available, but may be obtained from the corresponding author upon reasonable request.
